# Management practices in community-based HIV prevention organizations in Nigeria

**DOI:** 10.1186/s12913-021-06494-1

**Published:** 2021-05-22

**Authors:** David Akeju, Nerissa Nance, Andrea Salas-Ortiz, Ayoola Fakunmoju, Idoteyin Ezirim, Adejumoke G. Oluwayinka, Omoregie Godpower, Sergio Bautista-Arredondo

**Affiliations:** 1 Department of Sociology, University of Lagos, Lagos, Nigeria; 2 University of California, Berkeley, Berkeley, USA; 3 National Institute of Public Health, Mexico and University of York, York, UK; 4 National Agency for the Control of AIDS (NACA), Abuja, Nigeria; 5 Society for Family Health (SFH), Abuja, Nigeria; 6 Center for Health Systems Research, National Institute of Public Health, Universidad 655 Colonia Santa María Ahuacatitlán, Cerrada Los Pinos y Caminera C.P, 62100 Cuernavaca, Morelos Mexico

**Keywords:** Management practices, HIV prevention interventions, Female sex workers; community-based organizations, Nigeria

## Abstract

**Background:**

Nigeria has one of the largest Human Immunodeficiency Virus (HIV) epidemics in the world. Addressing the epidemic of HIV in such a high-burden country has necessitated responses of a multidimensional nature. Historically, community-based organizations (CBOs) have played an essential role in targeting key populations (eg. men who have sex with men, sex workers) that are particularly burdened by HIV. CBOs are an essential part of the provision of health services in sub-Saharan Africa, but very little is known about the management practices of CBOs that provide HIV prevention interventions.

**Methods:**

We interviewed 31 CBO staff members and other key stakeholders in January 2017 about management practices in CBOs. Management was conceptualized under the classical management process perspective; these four management phases—planning, organizing, leading, and evaluating—guided the interview process and code development. Data analysis was conducted thematically using *Atlas.ti* software*.* The protocol was approved by the ethics committees of the National Institute of Public Health of Mexico (INSP), the National Agency for the Control of AIDS in Nigeria (NACA), and the Nigerian Institute for Medical Research (NIMR).

**Results:**

We found that CBOs implement variable management practices that can either hinder or facilitate the efficient provision of HIV prevention services. *Long-standing* CBOs had relatively strong organizational infrastructure and capacity that positively influenced service planning. In contrast, *fledgling* CBOs were deficient of organizational infrastructure and lacked program planning capacity. The delivery of HIV services can become more efficient if management practices are taken into account.

**Conclusions:**

The delivery of HIV services by CBOs in Nigeria was largely influenced by inherent issues related to skills, organizational structure, talent retention, and sanction application. These, in turn, affected management practices such as planning, organizing, leading, and evaluating. This study shows that KP-led CBOs are evolving and have strong potentials and capacity for growth, and can become more efficient and effective if attention is paid to issues such as hierarchy, staff recruitment, and talent retention.

**Supplementary Information:**

The online version contains supplementary material available at 10.1186/s12913-021-06494-1.

## Background

Nigeria continues to experience one of the largest HIV epidemics in the world. As of 2019, 1.8 million people were living with the virus nationally [1]. Key populations (KPs), such as female sex workers (FSWs), are at particular risk of infection. In 2016, an estimated 14.4% of FSWs in Nigeria were living with HIV [2]. This population faces a myriad of challenges related to receiving care, including stigma, discrimination, and socioeconomic stressors [3–5]. Although HIV prevention and treatment services are largely subsidized in Nigeria, the structure for delivering service to KP (public/CBO clinics) and social norms that discriminate against vulnerable groups like FSW can inhibit these KPs from accessing them [6, 7]. Therefore, addressing this burden of disease and creating access to care necessitates engaging CBOs in the care delivery chain. CBOs take different forms and their functions vary by context. A CBO has been defined as “a public or private non-profit organization that represents a community or a specific part of a larger community, and targets meeting a specific need in that community” [8, 9]. In the context of this study, a CBO is a group of individuals with common interest in populations most at risk for HIV; their flexible organizational structure and rapport with vulnerable groups are key to implementing prevention services.

Evidence suggests that CBO involvement in the delivery of HIV services can significantly contribute to health services access and the reduction of HIV spread through stigma reduction and effective community mobilization and engagement [10–14]. The invaluable services that CBOs provide to communities are completely donor-driven and donor-funded. However, recent evidence shows that funds from international donors for HIV-related services in low- and middle-income countries have been shrinking [10, 15]. The significant decline in the funding of HIV programs has caused concern about sustainability of the funding model [1]. Despite scarcity, Nigeria has historically been one of the highest recipients of Global Fund financing [16]. Since the burden of HIV in Nigeria is high, the country will continue to play a significant role in the global efforts to eradicate HIV. Consequently, donors have emphasized the importance of optimizing the efficiency of existing program funding in order to make the most of limited resources [13, 16–18].

Efficiency can be achieved either by minimizing the number of inputs used to provide a health service or by maximizing the number of outputs. Optimization faces barriers in low-income health markets due to the rigidity of the labor market, scarce competition, and lack of skills and training [19–22]. Under such constraints, it is imperative to find levers that can increase efficiency. Management practices are tools or sets of general practices used by firms to achieve better outcomes [23]. The effective management of human, material, and financial resources is one potential way to optimize under constraints, and has been linked to increased productivity in firms and to efficiency in hospitals and local health facilities [20, 24–27]. Effective management practices can increase employees’ abilities to meet organizational goals [28], and align organizational objectives [29], which in turn increases overall productivity, and efficiency. Due to the importance of management, there have been concerted efforts to identify and measure management practices in health facilities [19, 24, 26, 29].

However, little evidence has been documented about the management practices of CBOs in Nigeria, other than the role they play in economic development [30]. Findings from other contexts like the United States may provide some insights into the management capacity of CBOs implementing HIV programs in other contexts. Two studies which focused on CBOs were conducted in the United States in 2008 and 2015. The former assessed the capacity of 20 CBOs implementing a HIV/AID program in 9 multicultural rural and urban communities, and the latter examined facilitators and barriers to effective project implementation among 72 CBOs working with men who have sex with men. Findings from these two studies showed that CBO management capacity to plan, implement, and evaluate success was very weak [31]. Additionally, CBO managers were not always mindful of the characteristics and skills required for effective leadership in program implementation, such as employing personnel who lacked the capacity for program implementation [17]. Irrespective of human resource capacity, organizational infrastructure is also crucial for efficient service delivery [32]. While these may be relevant to CBOs providing HIV services in Nigeria, the different contexts make direct comparison challenging. This study aims to fill the gaps in knowledge about management practices among CBOs providing HIV services to KP in Nigeria. Nigerian CBOs are unique because of the crucial role they play in reaching the KP with HIV preventive services, and for that reason are particularly ripe for management intervention.

## Methods

Using an ethnographic and content analysis framework, we conducted a qualitative investigation of management practices among CBOs in Nigeria [33, 34]. It also allowed us to engagement deeply with study participants and comprenhend the many structural and behavioral issues that could influence management practices among CBO managers. In the analysis, we segmented management practices into four interconnected phases namely: *planning*, *organizing*, *leading,* and *evaluating*, comprising 22 indicators of management practices (Supplementary Table 2). We defined the phases as follows: *planning* as identifying the goals and objectives of the organization and outlining activities, resources, and the timeline to achieve those objectives; *organizing* as establishing and maintaining the necessary relationships between human, material, and financial resources through resource allocation; *leading* as setting clear direction for individuals, groups, and the organizations; and *evaluating* as measuring whether organizational structures are working properly and identifying and addressing barriers as needed [35–37].

### Sample population

This study focused on managers working in CBOs that have received sub-award grants from an implementing partner. The implementing partner was a large non-profit organization was funded through the SHiPS for MARPs (Strengthening HIV Prevention Services for Most-at-Risk Populations) and the Global Fund Programs. Both funding programs focused on the provision of HIV testing and counselling (HTC), sexually transmitted infections treatment (STIT) and HIV education (HIVE) services to FSWs and other KPs. The goal of the intervention was to expand access to more high-risk populations to ultimately lower the HIV prevalence rates in these groups. The sampling frame was 31 CBOs spread across the north and south geopolitical zones, from which 7 were purposively selected based on their involvement in the implementation of HIV prevention projects for FSWs. Given the scale of insecurity in Nigeria which made some states in the south and the north inaccessible, sampling of CBOs was limited to Abuja and Nasarawa (in the north) and Lagos (in the south). For the purposes of this study, we focused on services offered by CBOs to FSWs only.

### Data collection

In-depth interviews at the 7 CBOs were conducted by the research team in January of 2017. Team members brought varied backgrounds that informed data collection. All questions, contained in an interview guide, were developed by authors based on study themes (Supplementary File 1). The method adopted for data collection enabled a direct interaction with: eight program officers, who were responsible for coordinating all daily activities and managing human resources within CBOs; seven monitoring and evaluation officers, who were tasked with the responsibility of ensuring that activities are carried out in line with the project design and process; two executive directors who provided overall leadership to the CBOs; and five volunteers who were non-permanent staff of CBOs recruited on ad-hoc basis to conduct HIVE, HTC, and STIT outreach activities. We also had face-to-face interactions with nine personnel from the implementing partners. Participants represented a variety of genders and educational backgrounds. The mean length of experience was 24.6 months; minimum length was 1 month and maximum length was 84 months. All interviews were conducted privately in the office spaces of the CBOs or implementing partners, were standalone, and lasted between 45 and 75 min in length. Interviews were digitally recorded, transcribed, and anonymized. Before commencing data analysis, preliminary findings were shared among CBO managers and implementing partners for feedback.

### Data analysis

Interviews were transcribed and reviewed for correctness and completeness. We generated a list of codes in line with the research themes. The majority of codes (*n* = 13; 59.1%) were defined a priori, though some codes (*n* = 9; 40.9%) emerged during analysis. To ensure inter-coder reliability, the codes were defined and mutually agreed upon by members of the research team. In order to ensure mutual understanding of the coding process between the research assistant and data manager, 10 (33%) of the transcripts were jointly coded, while the remaining transcripts were coded by a research assistant under the supervision of the data manager. Organization and generation of output were conducted primarily by the data manager and data were analyzed thematically using *Atlas.ti* software, v6.0 [38]. Data were analyzed in two stages. In the first stage, members of the research team categorized the analysis into identified themes. Results were shared among other members for collective review. In the second stage, members of the research team reviewed and refined findings by connecting themes and drawing further insight from the data. Our reporting is aligned with the *Consolidated Criteria for Reporting Qualitative Research* (COREQ) [39].

### Ethical approval and informed consent

The protocol for this study was approved by the Ethics Committee of the National Institute of Public Health, Mexico (CI-1403), the Health Research Ethics Committee of the National Agency for the Control of AIDS (NACA, FHREC/2016/01/58/08–08-16), and by the Nigerian Institute for Medical Research (NIMR, IRB/17/024). Written consent was obtained from all participants through the use of an informed consent form. Consent was also obtained from all participants to digitally audio record the interviews.

## Results

### Respondents socio-demographics

A total of 31 respondents participated in the interview. Slightly over half of the interviewees were female (*n* = 16, 52.0%). In terms of educational attainment, the sample was relatively evenly split between high school certificate (*n* = 10, 32.2%), university degree (n = 10, 32.2%), and postgraduate education (*n* = 9; 29.0%). A majority of respondents were program officers (n = 10, 32.3%), while others were employees of implementing partners (*n* = 8, 25.8%), evaluation officers (*n* = 6, 19.4%), or community facilitators (*n* = 5, 16.1%). Only a few respondents were in the category of executive director (*n* = 2, 6.4%). The mean age of respondents was 32.6 (standard deviation SD: 4.8 years; range: 24–48 years). Average experience (in months) for CBO managers (program officers, monitoring and evaluation officers, and community facilitators) was 24.6 (range: 1–84 months; Supplementary Table 1).

### Models of CBOs

**Fig. 1:**
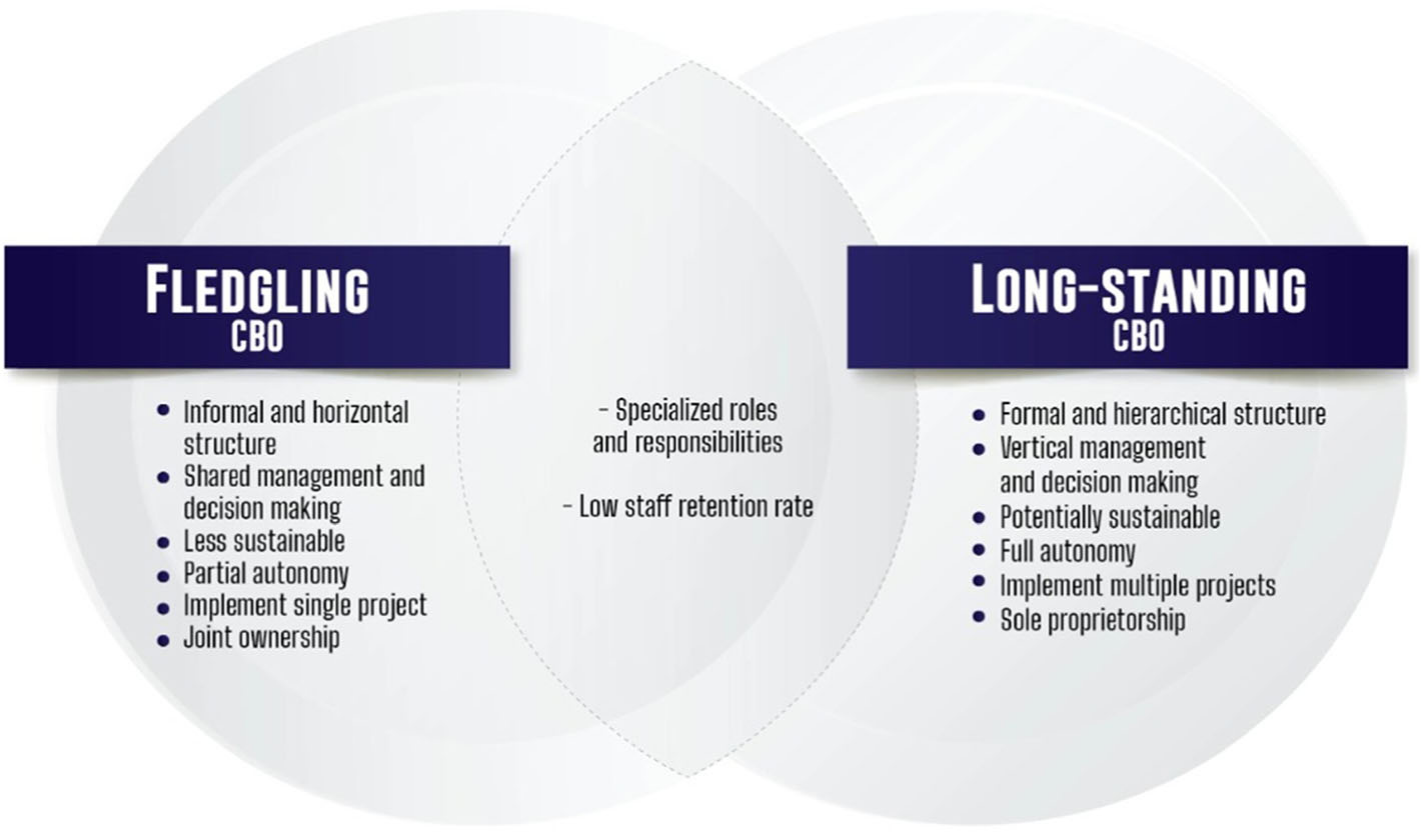
Divergence and convergence of characteristics among CBOs

We found that all CBOs operated heterogeneously around the three health prevention services (HIVE, HTC, and STIT), while still maintaining the procedure imposed by implementing partners. Within this context, we classified CBOs into two principal models, the *fledgling* and *long-standing* (Fig. 1)*.* These models differed by corporate status, degree of autonomy, formality, decision-making, sustainability, and ownership. Fledgling CBOs tended to be newer and were created by a cohort of friends who had long-standing relationships leading to a horizontal management structure among them. In contrast, long-standing CBOs tended to be older, comprised of individuals brought together by formal goals rather than by friendship.

The management process — divided into four mutually inclusive stages of planning, organizing, leading, and evaluating — was significantly influenced by the characteristics found among CBOs.

### Planning

In the planning phases, three key management issues influencing the provision of health services emerged, namely: *organizational infrastructure*, *planning autonomy, and staff recruitment*.

#### Organizational infrastructure

Organizational infrastructure constitutes CBOs’ policies and procedures on assigned roles and responsibilities of personnel. Results show that *long-standing* CBOs possessed some degree of organizational infrastructure and implementation capacity that significantly aided the planning phase of service delivery. These attributes also enabled them to integrate and coordinate available human resources around multiple activities. In contrast, *fledgling* CBOs lacked organizational infrastructure, had low organizational capacity, and relied largely on the external support of the implementing partner to plan activities and to deploy human and material resources. This meant that planning autonomy was largely impaired because planning and operational guidelines were imposed on CBOs by funding requirements – a situation which created a parallel planning structure for *long-standing* CBOs because they operated multiple projects. It also facilitated a complex procedure for managing human resources, especially because few personnel were in charge of running multiple projects, which often meant multi-tasking between projects. Since most *fledgling* CBOs had a single project to implement, they were not as encumbered with planning challenges as *long-standing* CBOs. Lack of autonomy among all CBOs meant that the implementing partner often interfered with the operational procedures, recruitment of personnel and volunteers, and planning budgets for CBOs. However, the degree of interference varied, and *fledgling* CBOs experienced greater interference than *long-standing* CBOs.

#### Staff recruitment

This was conducted relying on the social capital of older staff or other related individuals among *fledgling* CBOs, while *long-standing* CBOs mostly adopted formalized procedures for staff recruitment. These two methods gave rise to a mix of personnel with varied skills and competences. For example, among *fledgling* CBOs, skillsets among personnel were rather poor and personnel were prone to organizational challenges for which external support was required. A monitoring and evaluation officer at one of the *fledgling* CBOs recounted his recruitment procedure using informal networks:

*I was once a volunteer and I know a bit about it and I have the passion to work for the community … I met this program officer here because we use to have meeting together and we live in the same community. So, when he saw me he asked me what I was doing I told him “nothing for now”, then he said he could involve me in a project. That was how I came in to the organization.*

Monitoring and Evaluation Officer, Female, 12 months’ work experience, CBO #29

The recruitment procedure allowed CBOs to recruit a staff member who was “*doing nothing for now*” but knew “*a bit*” of what was done, and was “*passionate*” about what was being done. This procedure often led to loss of momentum in project management and implementation, as new staff with very low skills would have to rely on old staff or external partners to help them learn on the job. The loss of momentum resulting from this recruitment procedure would significantly impact on the capacity of CBOs to organize resources and personnel and retain talent.

### Organizing

Some of the indicators which measure how CBOs were organized includes how their personnel utilize available human and material resources for achieving organizational goals. Findings show that this component of the provision of services was significantly influenced by staff skillset, staff turnover, and financial autonomy.

#### Staff skillset and staff turnover

We found among *fledgling CBOs* that personnel were significantly deficient in expertise and skills for project implementation. Often, though, when a substantial level of skill has been acquired they would move to, as one interviewee called it, “greener pastures”— i.e. greater remuneration and/or a higher position at a larger organization. This created high rates of staff turnover which, in turn, led to depletion of personnel. While this was mentioned in several interviews, one monitoring and evaluation officer, referring to the way in which human resources were organized by CBO personnel, summarized the problem succinctly:

*I look at what is happening generally I look at the personnel, people that are providing the services, our human resource persons... could it be that is as a result of their poor performance? Could it be that is as a result of their incompetence? Could it be the community members? In every organization, there are internal control and they could be some kind of redeployment, [we recruit] these personnel, we know their capacity and we know their behavior, their level of interaction, attitude and their level of communication and how much they can actually impact. They are under our supportive intervention and mentorship all through like I said, if there is enough fund, we would have hired the best hands, the best experts but since this fund are not there to hire the best experts, like those with B.Sc.*

Monitoring and Evaluation Officer, Male, 24 months’ work experience, CBO #18

The effect of this on management of human resources was substantial. It was sometimes difficult for managers to coordinate activities effectively, owing to the mismatch in skills of newly employed personnel and tasks assigned. For effective organization of resources, there was evidence of skill exchange and collaboration with other agencies, particularly among *fledgling* CBOs, in order to enhance reach and impact service delivery. Such practices were not found among *long-standing* CBOs. Importantly, there were no mechanisms for sharing such creative and innovative ideas between these two models of CBOs.

#### Financial autonomy

We found that organizing human resources around goals and objectives was significantly influenced by CBOs’ lack of financial autonomy. This manifested in two ways. First, the funding model adopted made the implementing partner directly responsible for remunerating a high percentage of volunteers, which often caused volunteers to value or prioritize directives from the implementing partner over those from CBO managers. Sometimes, this hindered CBO managers’ efforts to deploy human and material resources for effective service delivery. It also impacted on the routine flow of work. A monitoring and evaluation officer expressed his frustration and provided further insight into how this structure of financing and remuneration created a bottleneck in management:

*I should be able to make a decision … . like [if I decide that] this is what should be done and this person said No, you can’t tell me what to do [because] [name of CBO officer] said this … It’s like this person does not have a clear job description, so to him it is [the implementing partner] that employed him, and not … [CBO]. As an M&E my job function that relates to him he’s less concerned*

Monitoring and Evaluation Officer, Male, 24 months’ work experience, CBO #18

We also found that expenditures were organized around a set of overhead costs at fixed amounts across these CBOs. Limiting CBOs’ expenses on recurrent overhead costs created a structure of expenditure that did not consider the peculiarity of and contexts of the CBOs, which may necessitate flexible expenditure pattern. While this rule applied to both models of CBOs, *fledgling* CBOs were most affected because they did not operate multiple projects. In contrast, *long-standing CBOs* were able to spread recurrent costs over multiple projects. The rigidity in expenditure patterns complicated financial accountability and expenditure for *fledgling* CBOs such that initiatives and effective deployment of human and financial resources were significantly affected.

### Leading/directing

One key management facet that influenced the leading and directing phase was shared decision-making. Roles and responsibilities of managers in setting clear direction and influencing others were specialized and not particularly different between the two CBOs. However, we observed a more vertical and rigid hierarchy among *long-standing* CBOs, and a flexible/horizontal approach among *fledgling* CBOs. The flexibility among *fledgling* CBOs promoted shared decision-making, inclusion, and team spirit, which consequently impacted on job satisfaction among personnel. While this flexibility was a hindrance in the planning phase, it was an asset in the leading and directing phase. The report below from one of the personnel further supports the point:

*The garbage can method is a kind of management method whereby everyone matters in an organization, even the gatekeeper, the cleaner, everyone brings a suggestion to the table, a suggestion is referred to as garbage, then the garbage is put in a can and then you shuffle it up or we iron it within ourselves and we see (laughs) the best decision to go with, if he is flexible I think it will have a positive effect on the project, that is it helps the project to move forward*

Case Management Officer, Male, 12 months’ work experience, CBO #15

In contrast, the hierarchical structure of responsibilities and decision-making among *long-standing* CBOs catalyzed more feelings of discontent and disconnection:

*Everything that comes into the organization has to pass through the executive director. … The structure is there it is just that it … . but not fully the way I want it to be. Number one, most times he [the ED] is hardly around and there are some decisions we have [to take] that has to depend on him and the things we need to get the work done is not available and it is quite frustrating*

Program Officer, Female, 24 months’ work experience, CBO #4

Managers of *long-standing* CBOs were sometimes unable to make decisions on important and urgent issues affecting service delivery because in most cases executive directors were not available to lead or direct. We also found that executive directors were less involved in the daily management of CBOs, which affected service delivery and sometimes led to delayed or unmet goals.

### Evaluating

Evaluation was an integral part of the provision of services. However, we found that *logistical challenges* and the use of *sanctions and rewards* affected service delivery during the evaluating phase.

#### Logistical challenges

Logistical challenges significantly hampered monitoring and supervision activities. Service delivery for CBOs was target-driven and the framework for evaluating performance was largely hinged on the ability of CBOs to meet these targets. This often heightened the pressure among personnel to perform, and because of shortfall in logistic support for monitoring and evaluation, volunteers sometimes resorted to dishonest and unethical practices to meet targets. In addition, poor communication and logistics caused some CBOs to experience overlap in demarcated territories. This overlap of service area meant efforts were duplicated or “double counted”, i.e. recorded or reported more than once. This likely increased project expenditure and affected optimal resource allocation. A point from one of the monitoring and evaluation officer provides more insight:

*What I mean by double counting is … , you have two different facilitators probably working on the same site. And when they get to that site probably one facilitator may not know that the other facilitator is already capturing a particular peer and that particular facilitator will capture that same peer and when it gets to me I will observe that we can’t be servicing the same person over and over again because we give refreshment to them we give item.*

Monitoring and Evaluation Officer, Female, 6 months’ work experience CBO #6

#### Sanctions and rewards

Regardless of evidence of some unethical practices and poor performance, very few CBOs applied sanctions. When sanctions were applied, such as through removing personnel or implementing salary deductions, it was usually preceded by corrective measures such as verbal or written warning. *Long-standing* CBOs applied this sanction more often than *fledgling* CBOs. The type of relationship among managers of these two kinds of CBOs – bonded, integrated, and informal among *fledgling* CBOs and structured and formal among *long-standing* CBOs – might have accounted for the relative difference in their application of sanctions. Applied sanctions applied existed in form of threats and salary deductions, as reported by one of the executive directors:

*Okay. Like when they are late in turning in their reports, like there was one incidence that happened that their salaries were reduced because they didn’t turn up their report, not just turning up their report but the sessions, the number of sessions they were supposed to have, they didn’t have up to that session so we now reported to [The implementing partner] and some part of their salary was deducted that time.*

Executive Director CBO, Female, 60 months’ work experience, # 26

While sanctions were rarely applied, a positive reward system existed across the two models of CBOs where personnel who performed well were rewarded in different ways, including promoting exceptional volunteers to the next vacancy.

## Discussion

Our study investigates management practices among CBOs providing HIV services to KPs in Nigeria. Findings from this study highlight several management issues that either hinder or facilitate the provision of HIV prevention services. These barriers and facilitators were replicated in both types of CBOs (*long-standing* and *fledgling*) to varying degrees. Reward for good performance, for instance, was a facilitator while rare application of sanctions was a barrier. When these divergent elements are present within the same CBO, it is difficult to know how the effect of one (facilitator) neutralizes the effect of the other (barrier). For example, *fledgling* CBOs were enthusiastic and passionate, yet suffered from a lack of formal organizational infrastructure. One potential remedy to this barrier may be knowledge sharing. *Long-standing* CBOs demonstrated a fair degree of organizational infrastructure, and this attribute might be transmissible to *fledgling* CBOs if opportunities were created for knowledge and experience sharing between the two models [40, 41].

Hierarchy was a major challenge among *long-standing* CBOs. The vertical structure of management had multiple layers which disrupted the flow of decisions from top to bottom. As was observed in this study, managers in organizations with hierarchical structure serve as liaisons to manage the gaps and translate expectations from upper management to frontline employees, which can lead to weak interactions between top management and frontline staff [42]. Thus, among most *long-standing* CBOs, field staff were not only disconnected from upper management, but communication between upper management and middle management was also fractured. This made decision-making cumbersome and often led to frustration among personnel [43, 44]. This barrier was potentially disruptive to the provision of services as important decisions that could facilitate effective and efficient service delivery were delayed. In contrast, *fledgling* CBOs had a flat structure which empowered staff to make a range of decisions on their own given the seamless flow of information from top down and from bottom up. Thus, hierarchical structure among *long-standing* CBOs did not encourage inclusion, commitment, and decision-making, but a flexible and flat structure, typically found among *fledgling CBOs*, inspired confidence, inclusion, and passion [45].

Enthusiasm and passion of staff facilitated effective service delivery among *fledgling CBOs*, supporting evidence that passion and commitment are related features of *emotional contagion* among personnel that can positively influence productivity [44]. These were the main management attributes that stood out among *fledgling* CBOs that contributed significantly to the provision of services. Enthusiasm and passion encourages teamwork, and highly motivated staff [43, 44, 46]. While this model of CBOs suffered organizational challenges, their lack of structure allowed for a greater degree of flexibility. A united workforce—where each employee has a good grasp of program goals and objectives—has positive effects on the organization. Teamwork can make more effective and efficient use of labor and can improve productivity by maximizing the different strengths and skills of team members so that a greater variety of tasks may be tackled [47]. It reduces workloads for all employees by enabling them to share responsibilities or ideas [43]. Studies on teamwork have shown that the more widespread teamwork is in an organization, the higher the level of organizational innovation [44]. In contrast, teamwork can also be accompanied by unwanted phenomena that result in performance loss or poor decision-making [46, 47]. While studies have shown the pros and cons of teamwork [43, 44], results from this study show that CBOs, particularly *fledgling* CBOs, can leverage this facet in order to promote inclusion, innovation, and improved service outcomes.

Having multiple projects, which was a main feature of *long-standing* CBOs, was instrumental in offsetting the organizational setback that lack of financial autonomy could cause. Sharing cost among projects helped *long-standing* CBOs to reduce the burden of rigid overhead costs. Decision-making is a key management issue that promotes growth, efficiency, and effective service delivery [43, 45]. Inability of *long-standing* CBOs to make decisions might have negatively impacted their potential for efficiency. In other words, were hierarchy to be flat and decision-making inclusive, service delivery might be more effective and efficient.

Rewarding performing staff is an effective means of motivating not just personnel that are productive, but a means of influencing those poorer performance [42, 48]. Financial rewards are by no means the only way to motivate. Non-financial rewards are also important in directing and shaping desired behaviors among employees [48, 49]. Service delivery among CBOs is target-driven and the ability to meet or surpass targets is often coupled with financial and non-financial rewards. Pay raises, verbal and written recognitions, awards and certificates, are some of the reward systems used by CBOs. Rewards can help to motivate personnel using reinforcement theory, which states that behavior can be reinforced by basic stimulus–response linkages [50]. In other words, when personnel are rewarded for desired behavior, they are more likely to behave that way in the future [49]. This aspect of management might account for why some CBOs would deliver on targets despite rare application of sanctions. It could also indicate that positive reward may have the potential to suppress behaviors that would otherwise necessitate the use of sanctions.

Another potential way to improve service delivery would be a more formal system for talent attraction and retention that could help both models of CBOs to achieve better results. The lack of deliberate policy geared towards the creation of a workforce heightened the depletion of human resources and slowed down the pace of work. Creating a workforce plan can help monitor strategies on the attraction and retention of talent [51, 52]. These strategies include recruitment, induction, performance management, professional development, and succession planning [32]. However, the two models of CBOs discussed in this study fall short of this and, as a result, they constantly experienced loss of momentum in management and project implementation. One of the features of such practice includes skill mismatch between tasks meant to be done and skills required to implement them, as well as frequent and high turnover rates of staff. Investment of time, financial, and human resources in retraining personnel could avoid regular interruption in service delivery.

### Limitations

This study aims to understand management practices among KP-led CBOs spread across Nigeria. Due to security concerns in the country and resulting transportation challenges, we could not sample from all six geopolitical zones of the country. Our sample was geographically limited to Abuja, Lagos, and Nassarawa states, and therefore disproportionately reflects views from urban and suburban CBOs. These CBOs may face different challenges than more rural CBOs. We also focused primarily on KP-led CBOs and only on those serving FSW. Therefore, findings from this study might not be generalizable to CBOs serving other populations or that focus on services other than HIV transmission prevention.

## Conclusions

This qualitative study highlights the importance of management practices in efficient delivery of HIV services to KPs by CBOs in Nigeria. CBOs providing HIV services to KPs can become more efficient and effective if attention is paid to issues such as hierarchy, organizational infrastructure, staff recruitment procedure, talent retention, and knowledge sharing between the different CBO models. The analysis also reveals that, far from being a monolith, the practices of management at each model of CBO are diverse. It is key for funding entities to keep this in mind as they continue to support CBOs, since they are uniquely positioned to support facilitators and discourage barriers to productivity. Likewise, it is important that future research examining or intervening on management practices at CBOs account for the heterogeneity of management implementation. At the same time, CBOs are evolving and have a capacity for growth that is ripe for intervention.

## Supplementary Information

**Additional file 1: Supplementary Table 1**. Sociodemographic information of respondents. This Table shows the sociodemographic description of participants.

**Additional file 2: Supplementary File 1.** Guide for semi-structured interviews. This File shows the tools used for data collection.

**Additional file 3: Supplementary Table 2**. Code definitions and frequencies. This Table shows the definition of the codes analyzed and frequency of appearance.

## Data Availability

The data collected and analyzed during the current study is available from the corresponding author, upon request.

## Abbreviations

CBOs: Community Based Organizations
FSWs: Female Sex Workers
HIV: Human Immune Virus
HIVE: HIV Education
HTC: HIV Testing and Counseling
INSP: National Institute for Public Health
KP: Key Population
NACA: National Agency for the Control of AIDS
NIMR: Nigerian Institute for Medical Research
SHiPS for MARPs: Strengthening HIV Prevention Services for Most-at-Risk Populations
STIT: Sexually Transmitted Infections Treatment
