# Supplementary material for: Management practices in community-based HIV prevention organizations in Nigeria
Source: BMC Health Serv Res. 2021 May 22;21:489. doi: 10.1186/s12913-021-06494-1 (PMC8141130; doi:10.1186/s12913-021-06494-1)
Supplement: Supplementary file 1 — Additional file 1: Supplementary Table 1. Sociodemographic information of respondents. This Table shows the sociodemographic description of participants. [file 12913_2021_6494_MOESM1_ESM.docx]

**Supplementary Table 1. Sociodemographic information of respondents**

| **Socio-demographic variable** | **Frequency (%)** |
| --- | --- |
| **Gender**  Female  Male | 16 (52.0%)  15 (48.0%) |
| **Total** | **31** |
| **Education**  High school  Bachelor of Science  Postgraduate certificate  Less than high school  Ordinary National Diploma | 10 (32.3%)  10 (32.3%)  9 (29.0%)  1 (3.2%)  1 (3.2%) |
| **Total** | **31 (100.0%)** |
| **Job title**  Program officer  Employees of implementing partners  Monitoring and evaluation officer  Community facilitator  Executive director | 10 (32.3%)  8 (25.8%)  6 (19.4%)  5 (16.1%)  2 (6.4%) |
| **Total** | **31 (100.0%)** |
| Age  Number of observations  Mean  Minimum value  Maximum value  **Standard deviation** | 31  32.6  24  48  **4.82** |
| **Staff experience (in months)** | |
| **CBO manager**  Number of observations  Mean  Minimum value  Maximum value | 21  24.6  1  84 |
| **Employees of implementing partners**  Number of observations  Mean  Minimum value  Maximum value | 8  54.0  24  84 |
| **Executive Directors**  Number of observations  Mean  Minimum value  Maximum value | 2  120  108  132 |
