# Supplementary material for: Management practices in community-based HIV prevention organizations in Nigeria
Source: BMC Health Serv Res. 2021 May 22;21:489. doi: 10.1186/s12913-021-06494-1 (PMC8141130; doi:10.1186/s12913-021-06494-1)
Supplement: Supplementary file 2 — Additional file 2: Supplementary File 1. Guide for semi-structured interviews. This File shows the tools used for data collection. [file 12913_2021_6494_MOESM2_ESM.docx]

**Supplementary File 1. Guide for semi-structured interviews**

Costs, efficiency and the role of management in

HIV prevention interventions for female sex workers in Nigeria

**Qualitative component - Formative research**


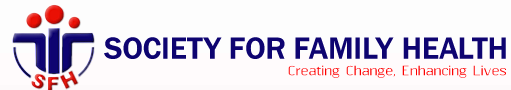


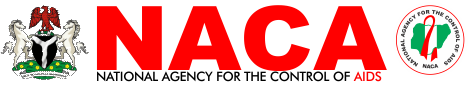


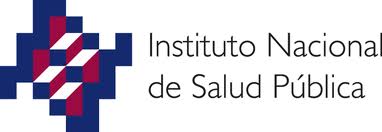


**CBO Managers: Semi-structured interview guide**

General information of the interview:

- Interview number
- CBO’s name
- State’s name
- Municipality name
- Date of the interview (DD-MM-YYYY)
- Facility type
- Facility administrator
- Gender of the interviewee
- Function/position at the facility
- Number of years in position
- Facility hours/weekly schedule

**Directions:** The bolded questions below outline main categories of questioning. The sub bullets detail topics for further probing. There is no need to ask every question, but rather to focus on the areas in which the respondent has the most to say. Remember to introduce the project and reinforce that all responses will remain confidential.

**Tell me about your work at the facility.**

- Why did you decide to work here?
- What is your day-to-day work like? Is this similar to others?
- Tell me about a time when you recently made a decision that impacted the facility.
- What type of relationship do you have with the other employees? Do they ever influence what you do or vice versa? (if respondent says yes, probe for a story)
- What are you most proud of from working here? Tell me more about that.
- What was the most difficult moment you experienced when working here? Tell me more about it.
- What’s your own personal career goal? What position would you like to have 2-3 years from now? What needs to occur for you to achieve that?

**What was it like to complete the journal exercise?**

- Looking back on it, were you surprised by anything you noted down?
- Were the decisions and activities you wrote down typical or were they out of the norm?
- I’m very interested by section X. Can you tell me more about that moment?

**Tell me about how the facility is managed. What are the most important decisions that are made? Who makes them?**

- What are the most important decisions that staff here make that influence the functions of the facility?
- Who are the key decision makers? Do they act alone or do others influence them?
  - If so by whom?
  - Can you think of a time where a nurse, patient, or other person involved in the care interaction influenced the facility to change a practice? Is that common? What would prevent another staff from doing the same?
- In what areas, do you think the facility is doing really well? What areas could be strengthened?
  - Why do you think those areas are weaker? Do you think others agree?
- If you could change anything, what would you change?

**What types of services does the facility deliver to FSW? Is there anything special or different that comes into consideration when serving this type of patient?**

- Can you tell me about a time when you were creating a new service or programs for FSWs? What types of decisions did you need to make? How did you and your colleagues approach them?
- Is anything harder about serving these types of patients?
- Compared to all of the services the facility delivers overall, what percentage is related to FSW?
- Why do you think FSW choose to visit this facility instead of others?

**What’s the most commonly delivered preventative service provided to FSW?**

- How often does the facility deliver this service on a daily basis?
- Walk me through the steps from the eyes of the patient. What do they need to do receive this service? Who do they interact with? What procedures must they complete?
- Are there any additional steps that come to mind from the perspective of the nurse or doctor? What steps must they take to provide this service?
- Is there anything about this process that you would like to change if you could?
- What do you think FSW patients are most satisfied with? What are they least satisfied with? Why does that occur? --tell me more about that.

**How long do staff members typically stay at this facility? Do they work here for many years or gain a little experience and move on to another opportunity?**

- Why do you think staff stay for so long?
- Why do staff members leave? Tell me about the last time a staff member moved onto another opportunity. Why do you think he/she left?
- Are there opportunities for staff to take on more senior roles or make more important decisions? Does that occur often?
- How does the facility decide which staff are successful at their duties? How do they recognize or reward those that do well?

**Tell me about the data that’s used in decision-making.**

- What type of data does the facility generate for its own internal use? Do you ever reference it? What do you do with the information? How is this data collected?
- Can you tell me about a time when you learned something interesting or surprising about the facility based on data that was collected internally?
- Is any of this information shared with others outside of the facility? How is it used? Can you tell me about a time when the facility shared some its data externally and something occurred as a result of it?
- What data is the least useful?
- What additional data might be helpful to have? What would you do with it?
- What types of technology are used to record and store the data? Do you ever reference old data? Why?

**What’s your favorite way to receive information about the facility? Can you tell me about a time when you used data to change something in the facility?**

- What’s the best format to receive data? For example, do you like paper reports, in-person briefings from those that collected the information, digital copies, etc.?
- What’s the most common format that you typically receive information? How come?

**How do you think this facility compares to other facilities that provide similar services?**

- What does this facility do well that others don’t?
- Is there anything you’ve seen in other facilities that you think this one should implement?
- Have you ever used data from another facility for decision-making here?

**Thank you so much for taking the time to speak with me. Is there anything else you’d like me to know?**

Costs, efficiency and the role of management in

HIV prevention interventions for female sex workers in Nigeria

**Qualitative component - Formative research**


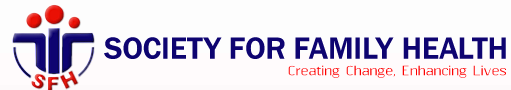


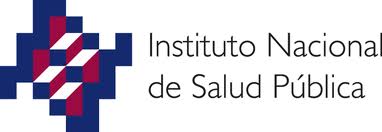


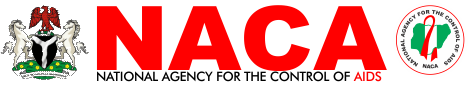


GUIDE FOR SEMI-STRUCTURED INTERVIEWS

**STAFF OF IMPLEMENTING PARTNERS**

**Directions:** The bolded questions below outline main categories of questioning. The sub bullets detail topics for further probing. There is no need to ask every question, but rather to focus on the areas in which the respondent has the most to say. Remember to introduce the project and reinforce that all responses will remain confidential.

**Prompt:** *The following guide consists of several sections that might be answered by different actors at the National Agency of for the Control of Aids (NACA) and The Federal Ministry of Health (FMoH) at Nigeria which have direct contact/communication with the Society of Family Health (SFH) staff in charge of HIV health services delivery and from SFH headquarters.*

*Thank you very much for agreeing to participate in this interview. My questions will be related to general aspects of the organization of HIV prevention activities at SFH. If you have any questions at any time during the interview, please ask me.*

General information of the interview:

- Interview number
- State’s name
- Date of the interview (DD-MM-YYYY)
- Gender of the interviewee
- Function/position at NACA
- Number of years in position
- Facility hours/weekly schedule

**Tell me about NACA and your work with them.**

- How long have you had your current position? How did you get it? What were you doing before?
- What do you like about your work? What makes you proud?
- What is your own personal career goal? Where do you see yourself in 5-10 years?
- Tell me about a time when you recently made a decision that impacted SFH operations. Why did you make it, and how? Can you tell me more about that?
- What type of relationship do you have with the staff at SFH? Do they ever influence what you do or vice versa? (if respondent says yes, probe for a story)
- Describe your last interaction with an SFH staff. What was that like? Can you tell me more? (Same question for CBO staff)
- Could you describe us the general HIV prevention activities that are carried out towards Female Sex Workers (FSW) in Nigeria?
- Could you describe us the procurement process carried out by the NACA/Ministry of Health for prevention supplies? Who provides them? Where do they go?
- How does the purchase of prevention supplies take place? What is the role of the NACA/FMoH?
- Are there any monitoring or evaluation of these processes? Who is in charge of it? How does it work?

**Tell me about how NACA operates. What are the most important decisions that are made regarding the CBOs? Who makes them?**

- Is there any coordinating unit in your agency dedicated to working with the CBOs?
- What information systems do you use related to HIV prevention activities?
- Who is in charge of updating these systems?
- How are CBOs connected to the overall goal of NACA? What about specifically for FSW services?
- Who has access to these systems or is there any kind of information that is restricted?
- How do you use the information in these systems? (Decision making, recommendations to coordination, SFH etc.)
- How do you monitor these information systems?
- Do community-based organizations/SFH have other information systems than NACA/FMoH? If so, could you describe them?

**Tell me about the data that’s used in the monitoring of CBOs.**

- Who is in charge of the monitoring and evaluation of SFH HIV prevention activities? How is the data collected?
- In general, how does NACA monitor SFH HIV prevention activities?
- **How is monitoring information used by NACA? By SFH? By the CBOs?**
- Has the data ever been used to make a decision? If so, what and how?
- What is the relationship between NACA/FMoH and the SFH headquarters regarding monitoring and evaluation of SFH HIV prevention activities?
- Describe the last monitoring session that took place. Can you expand on that?
- Are there any information systems used for the monitoring and evaluation process?
- What kind of external supervisions are done to the HIV prevention activities?
- What kind of information is mandatory for the HIV prevention activities to send to NACA/FMoH?
- Are there any sanctions for deficiencies? What about incentives?
- Are there any sanctions for wrong practices or poor performance? How common are these sanctions in providers of HIV prevention activities? What about incentives for good performance?
- Is there a mechanism for patients to report complaints and suggestions? If so, who is in charge for monitoring and follow up? What kind of follow up is given?

**When a FSW is diagnosed with HIV, how does the referral process work? The treatment process?**

- Is there a classification of the HIV/AIDS treatment services that NACA/FMoH uses? Is this classification based upon staff number, the number of HIV/AIDS patients attended or kind of services offered? Could you describe it?
- What is the relationship between NACA/FMoH and the HIV treatment services?
- Regarding HIV/AIDS treatment in Nigeria, is there any medicine/drug that is not free for patients/FSW? What about laboratory tests?
- Are there national guidelines or regulations about the correct filling of HIV clinical records?
- Is there a mechanism for patients to report complaints and suggestions? If so, who is in charge for monitoring and follow up? What kind of follow up is given?
