# Supplementary material for: Management practices in community-based HIV prevention organizations in Nigeria
Source: BMC Health Serv Res. 2021 May 22;21:489. doi: 10.1186/s12913-021-06494-1 (PMC8141130; doi:10.1186/s12913-021-06494-1)
Supplement: Supplementary file 3 — Additional file 3: Supplementary Table 2. Code definitions and frequencies. This Table shows the definition of the codes analyzed and frequency of appearance. [file 12913_2021_6494_MOESM3_ESM.docx]

**Supplementary Table 2. Code definitions and frequencies**

| **Management Phase and codes (n)^a^** | **Definition** |
| --- | --- |
| **Planning:** To identify the goals and objectives of the organization, so as to identify the activities, resources and the timeline to achieve those objectives | |
| 1. Organization Target Setting (130) 2. Targets feelings (101) 3. Target setting (105) 4. Communication (26) 5. Budgeting (3) | 1. The ability to set a CBO mission and vision just at the organizational level 2. Feelings and thoughts about how targets are used and operate, both individual and organizational 3. The extent to which staff understand well what needs to be completed in order for them to reach their goals 4. Communication with partners to carry out service provision activities. (To transmit messages from a sender to a receiver, in the context of service provision within CBO staff) 5. To make a financial plan according to the targets of the CBO |
| **Organising:** To establish and maintain the necessary relationships between human, material and financial resources by indicating which resources are to be used for specific activities and when, where and how they will be used | |
| 1. Retaining talent (14) 2. Attracting talent (18) 3. Continuous improvement (119) 4. Organization of workspace (9) 5. Input Organization of supplies (15) 6. Horizontal or ad-hoc power dynamics (16) 7. Team Work (42) 8. Organizational Challenges and barriers (254) 9. Service implementation (45) 10. Collaboration (23) | 1. Act of purposely keeping staff or attempting to keep staff because of their good performance 2. Act of purposely seeking out people highly skilled for the position they fill 3. Any act that seeks to improve processes already in existence at the CBO to make them faster, better, or more effective 4. The act of changing the physical workspace in order to make work better or easier 5. Evidence regarding an input stock organization system. 6. Management style that is lateral or cooperative; one staff member does not seem to have power over others 7. Implementing approach that recognizes the importance of everyone’s roles in achieving organizational goal 8. Difficult issues that impede or impact negatively on field and organizational operation 9. Description of how activities are implemented, according to the plans. (To arrange through systematic planning all the necessary activities to carry out services) 10. Planning or working with other organizations in other to achieve organizational goals. (Any action, process or activity carried out or that relies on support from other organization) |
| **Leading:** To set a clear direction for individuals, groups and the organization. The leader(s) must have the capacity to influence, nudge and inspire others to achieve the common goals | |
| 1. Interpersonal relationships (69) 2. Organizational hierarchy (13) 3. Motivation (24) | 1. Relationships or bonds between parties, usually co-workers but also manager-supervisee or with clients as well 2. Management style that is tiered and vertical; one staff member has clear power over others work 3. Internal or external factors/feelings that stimulate people to do something. (Actions or feelings examples of continuous encouragement) |
| **Evaluating:** To ensure that the processes and organizational structures are working properly. It also entails identifying barriers and tackling them | |
| 1. Rewarding high performers (15) 2. Removing poor performers (8) 3. Performance tracking (63) 4. Sanctions (8) | 1. Rewards or the act or rewarding any staff member or volunteer for their service 2. Any act of firing, demoting, or removing a poor performing staff member from their work 3. The measurement and recording of performance measures for staff or CBO 4. Negative incentives that are meant to punish staff for poor behavior, incomplete/poor work, etc. |

**^a^n = number of times the code was used during transcript coding**
